# Predictors of adherence to a digital health application in patients with inflammatory arthritis: retrospective analysis

**DOI:** 10.1007/s00296-025-06017-9

**Published:** 2025-12-15

**Authors:** Dmytro Fedkov, Christine Peine, Felix Lang, Abdullah Khalil, Johannes Knitza, Jan Leipe

**Affiliations:** 1 Department of Internal Medicine #3, Bogomolets National Medical University, Kyiv, Ukraine; 2 Medical Center Medical Clinic Blagomed LLC, Kyiv, Ukraine; 3 Midaia GmbH, Heidelberg, Germany; 4 Institute for Digital Medicine, University Hospital of Giessen and Marburg, Philipps University Marburg, Marburg, Germany; 5 Université Grenoble Alpes, AGEIS, Grenoble, France; 6 Rheumatology Department, Universitätsklinikum Schleswig- Holstein, Kiel Campus, Kiel, Germany

**Keywords:** Rheumatoid arthritis, Spondyloarthritis, Psoriatic arthritis, Digital health, Adherence

## Abstract

Digital health applications (DHAs) can support self-management in inflammatory arthritis (IA), but sustained adherence remains challenging in real-world use. To determine demographic and clinical predictors of 12-week adherence to a rheumatology-specific mobile health application in patients with IA. We retrospectively analyzed 2,036 adult users of the CE-certified Mida Rheuma App (2022–2024). Adherence was defined as self-reported body weight entry at week 12. Candidate predictors included age, gender, time since diagnosis, baseline body weight, and two patient-reported outcomes (PROs): Patient’s Global Assessment of Disease Activity (PGADA) and Patient’s Global Assessment of Pain Intensity (PPAIN). A Random Forest classifier (six predictors) was evaluated with repeated stratified 5 × 2 cross-validation; performance was summarized by receiver-operating characteristic area under the curve (ROC AUC). Of 2,036 patients, 526 (25.8%) met the adherence criterion. Adherence increased with age (peak ≈ 47.4% at 63–69 years) and showed a threshold with disease duration (peak ≈ 45.2% at 3,001–3,500 days, > 3,000-day inflection). PROs exhibited non-linear (“inverted-U”) patterns, with higher adherence at moderate-to-high symptoms (e.g., PGADA 35–40 ≈ 39.3%; PPAIN 10–15 ≈ 39.3%) and lower adherence at minimal or extreme values. Baseline body weight contributed modest predictive information, while the gender effect was minimal. The model achieved an ROC AUC of 0.627 (95% CI, 0.600–0.654), indicating modest but informative discrimination. In this large real-world IA cohort, 12-week adherence was highest among older patients, those with longer disease durations (> 3,000 days), and those reporting moderate-to-high symptom burdens. These results support the use of stratified onboarding and tailored support, particularly for younger or newly diagnosed patients and those at symptom extremes, and motivate the development of multidimensional adherence metrics in prospective studies.

**Supplementary Information:**

The online version contains supplementary material available at 10.1007/s00296-025-06017-9.

## Introduction

Digital health applications (DHAs), particularly mobile applications, continue to transform the way rheumatic patients manage their care [1]. They enable themselves to monitor their symptoms, to receive personalized recommendations, track their health status between medical visits, and increase their self-awareness and responsibility for their well-being [2–4]. In rheumatology, especially in rheumatoid arthritis (RA), spondyloarthritis (SpA), and psoriatic arthritis (PsA), DHAs focus and facilitate the collection of patient-reported outcomes (PROs), such as pain levels (e.g., visual analog scale (VAS)), disease activity indices (e.g., Routine Assessment of Patient Index Data 3 (RAPID-3), Bath Ankylosing Spondylitis Disease Activity Index (BASDAI), and quality of life measures [5–8].

While clinical trials demonstrate the potential of DHAs to reduce disease activity and improve self-management indicators, real-world evidence on long-term use is limited. Current evidence demonstrates that although initial engagement is often high, it tends to decline rapidly, typically within the first 4–8 weeks [11–14]. This undermines the overall effectiveness of mobile tools and questions their cost-effectiveness.

Adherence to digital tools is influenced by a wide range of factors, including demographic, clinical, psychological, and behavioral [15–18]. For example, older adults may have lower digital literacy [19] but often demonstrate higher adherence due to personal motivation and consistent routines [20–23]. Disease duration is one of the key predictors of established self-monitoring habits. Patients with a longer history of disease tend to have greater awareness of their condition and are more likely to follow recommendations [24–28].

In rheumatology, few studies have analyzed real-world usage patterns of DHAs in large cohorts. Most published works focus on the use of electronic PROs within clinical trials or as components of telehealth initiatives [29–32]. These studies have reported a tendency for better adherence among older patients, those with longer disease duration, and those receiving structured onboarding support.

However, gaps remain in understanding which demographic characteristics are associated with sustained adherence to DHAs in real-world rheumatology populations.

Identifying predictors of long-term adherence is essential for developing adaptive, stratified implementation strategies aimed at minimizing early disengagement and maximizing the utility of DHAs [33–36].

We therefore aimed to determine demographic and clinical predictors of 12-week adherence to a digital health application among patients with RA and SpA, including PsA, using real-world data from more than 2,000 users.

## Materials and methods

### Study design and patients

This retrospective observational study analyzed anonymized real-world data from 2,566 patients with inflammatory arthritis (RA, SpA, PsA) who used the CE-certified Mida Rheuma App (Midaia GmbH, Germany) between January 2022 and December 2024. The app offers personalized therapeutic and educational modules, including nutrition and weight-management content, physical activity and psychological coaching, sleep and stress hygiene, and medication literacy. All enrolled users have access to structured educational action plans. Details of the DHA have been described previously in more detail [37]. Briefly, the Mida Rheuma App enables patient monitoring using ePROS and offers personalized therapeutic and educational content. Inclusion criteria were: adult patients (≥ 18 years) with a diagnosis of RA, spondyloarthritis SpA, or PsA confirmed by a rheumatologist; consent for data use for research purposes; and availability of baseline data. Patients with complete records of baseline data were included for analysis of digital adherence.

Our primary adherence proxy was the presence of a week-12 weight entry, chosen for its objectivity and uniform availability. To contextualize this measure, we report concurrent adherence signals (e.g., counts of completed modules/assessments) showing higher activity among adherent users. To contextualize this unidimensional proxy, we additionally summarized concurrent app-adherence indicators (e.g., counts of completed therapeutic modules/assessments). We observed that these indicators were markedly higher in the adherent group. These adherence indicators were not used as predictors in the primary model to avoid circularity; however, they are presented descriptively to support construct validity (see Supplementary Table S1). Patients with available baseline data but missing body weight entry at week 12 were considered non-adherent.

The study was conducted in accordance with the ethical principles outlined in the Declaration of Helsinki (October 2024 revision) and the Good Clinical Practice guidelines. It was approved by the Local Ethic Committee of Medical Center Medical Clinic Blagomed LLC (protocol #107/5, dated August 13, 2022). All patients provided informed consent before using the Mida Rheuma App.

### Assessments

We analyzed a set of demographics, clinical, and patient-reported parameters to explore their association with 12-week digital adherence. The collected variables included demographic parameters (age in years, gender, and time since diagnosis in days), clinical characteristics (height in centimeters and baseline body weight in kilograms), and several patient-reported outcomes. These self-reported measures included the Patient’s Global Assessment of Disease Activity (PGADA) [38] and Pain Intensity (PPAIN) [39] (0–100 mm) in all IA patients. When available, RAPID3 [40] was recorded on its original 0–30 scale (all IA patients) and interpreted using standard categories: near remission (0–3.0), low (3.1–6.0), moderate (6.1–12.0), and high (12.1–30.0). For SpA, BASDAI [41] (0–10) and BASFI [42] (0–10). The Mediterranean Diet Adherence Screener (MEDAS) [43] and the total score from the Short Form Health Survey 36 (SF-36) [44] quality of life questionnaire, where available, were recorded for all IA patients.

### Statistical analysis

We aimed to identify predictors of adherence to a digital health intervention over a 12-week period. Initially, we applied multivariable logistic regression models; however, the inclusion of clinical scores, such as RAPID3 or BASDAI, led to convergence failure or perfect separation. This limitation rendered conventional regression techniques unstable for extended models.

To address this, we tested three modeling strategies: (1) a simplified multivariable logistic regression including three demographic predictors (age, gender, disease duration), (2) regularized logistic regression using L2 penalty (Ridge), and (3) a non-parametric Random Forest (RF) classifier.

The final RF (400 trees, class weight-balanced, minimum samples per leaf = 5) was trained in the Weight-Baseline cohort (*n* = 2,036). Predictors were: Days Since First Diagnosis Date, Weight Baseline, Age, PPAIN, PGADA, and Gender (1 = female, 0 otherwise. Model reporting followed Transparent Reporting of a multivariable prediction model for Individual Prognosis Or Diagnosis (TRIPOD)-aligned elements. Performance was estimated via repeated stratified 5-fold cross-validation (2 repeats) with out-of-fold predictions: the Receiver Operating Characteristic Area Under the Curve (ROC AUC) was 0.627 (95% CI 0.600–0.654), the Precision-Recall Area Under the Curve (PR AUC) was 0.359, and the Brier score was 0.189. We report performance at *p* = 0.50, and Youden’s threshold (~ 0.26), including Sensitivity, Specificity, positive predictive value (PPV), negative predictive value (NPV), F1, and Balanced Accuracy (Supplementary Tables 2 and 3). Calibration, Precision–Recall, and decision-curve analyses are shown in Supplementary Figs. S1–S3. To mitigate class imbalance (25.8% adherent), we used class weighting and examined decision thresholds.

For model interpretability, we prioritized permutation importance (Δ performance under feature shuffling) and SHAP (TreeExplainer) summaries. A global SHAP beeswarm ranks features by impact (Supplementary Fig. S5); SHAP dependence plots illustrate nonlinearity/thresholds for disease duration and age (Supplementary Figs. S6–S7). *(Impurity-based RF importances were not used as the primary metric.)*

To explore potential nonlinear associations descriptively (independent of the RF), continuous predictors were binned as follows: disease duration into 10 equal-width bins of 500 days (0–5,000); age and weight into deciles (quantiles); PtGADA and PPAIN into 5-point bins (0–5, 6–10, …, 95–100). These binned plots are descriptive; the RF captures nonlinearity without relying on cut-points.

Missing data handling. Because missingness likely includes both MAR (e.g., PRO gaps associated with observed age, disease duration, and adherence) and MNAR (exclusion due to absent baseline weight at onboarding), we used median imputation for model fitting and complemented inference with robustness checks (binning, permutation importance, and SHAP). Given probable MNAR among non-engagers, multiple imputation was not applied to avoid model-based extrapolation; we note this for prospective sensitivity analyses.

All descriptive and comparative statistics (e.g., t-tests, chi-square tests) were conducted in Python 3.10 (pandas v2.2, SciPy v1.11). A two-sided p-value < 0.05 was considered statistically significant.

## Results

Analysis was based on data from 2036 patients (1168 RA, 868 SpA [291 with PsA], of whom 526 (25.8%) self-reported body weight entry at week 12, and were hence classified as adherent. The remaining 530 (20.7%) lacked a baseline weight and were excluded. Because this entry is prompted at onboarding, its absence likely reflects limited early engagement with the app, suggesting informative missingness (consistent with an MNAR mechanism).

Table 1 presents the baseline demographic and clinical characteristics of adherent versus non-adherent patients. The mean age of the patients was 49.3 ± 13.6 years, and 77.8% were female (1582 females, 440 males, and 14 individuals of other genders). The average time since diagnosis was 1,699 ± 2,227 days, with a significantly longer disease duration in the adherent group (*p* < 0.001). Baseline body weight was significantly higher among adherent patients (78.9 ± 17.6 kg) compared to non-adherent (76.7 ± 17.8 kg, *p* = 0.006).

**Table 1:** Baseline demographics and ePROs in adherent vs. non-adherent users (n per variable shown in the first column)

Baseline variable, *n* = assessment available	Adherent (*n* = 526)	Non-adherent (*n* = 1510)	*p*
Gender (Female %), *n* = 2036	76.8%	78.0%	0.850
Age, *n* = 2036	49.2 ± 12.9	44.4 ± 12.8	< 0.001
Days Since First Diagnosis, *n* = 1700	3629.5 ± 3299.9	2890.1 ± 3513.1	< 0.001
SF36 Total, *n* = 226	53.5 ± 21.0	56.0 ± 22.9	0.466
Height, *n* = 2036	169.6 ± 12.5	169.2 ± 13.7	0.530
Weight, *n* = 2036	79.1 ± 19.2	78.3 ± 18.8	0.405
MEDAS Score, *n* = 526	6.1 ± 2.1	5.7 ± 2.5	0.251
BASDAI, *n* = 799	5.0 ± 1.8	5.1 ± 2.0	0.274
BASFI, *n* = 221	4.0 ± 2.3	4.0 ± 2.5	0.819
RAPID3, *n* = 1518	11.8 ± 5.5	13.0 ± 5.2	< 0.001
PGADA, *n* = 1959	54.3 ± 25.1	59.3 ± 25.2	< 0.001
PPAIN, *n* = 1959	49.8 ± 25.9	54.8 ± 25.1	< 0.001

Patient-reported outcomes, such as PGADA and PPAIN, were available in 1,959 patients, and RAPID3 was available in 1518 patients, with small but statistically significant differences between groups (Table 1).

Other variables, such as height, SF-36 score, and dietary quality (as measured by MEDAS), showed no significant differences. The proportion of female patients did not differ significantly between groups.

Among the tested models, the RF classifier demonstrated the most robust performance, with an ROC AUC of 0.627 in the final configuration, indicating moderate discriminative ability. At *p* = 0.50 the operating characteristics were: sensitivity = 0.131, specificity = 0.938, F1 = 0.200, Balanced Accuracy = 0.534; at Youden’s J (≈ 0.26): sensitivity = 0.627, specificity = 0.580, F1 = 0.443, Balanced Accuracy = 0.604 (Supplementary Tables S2–S3). It offered both resilience to missing data and interpretable importance rankings of variables.

Adherence indicators covaried with the adherence proxy: adherent users exhibited substantially higher completion rates of therapeutic modules/assessments relative to non-adherent users (Supplementary Table S1). This pattern supports the face/construct validity of using week-12 body-weight entry as a marker of sustained use.

**Fig. 1:**
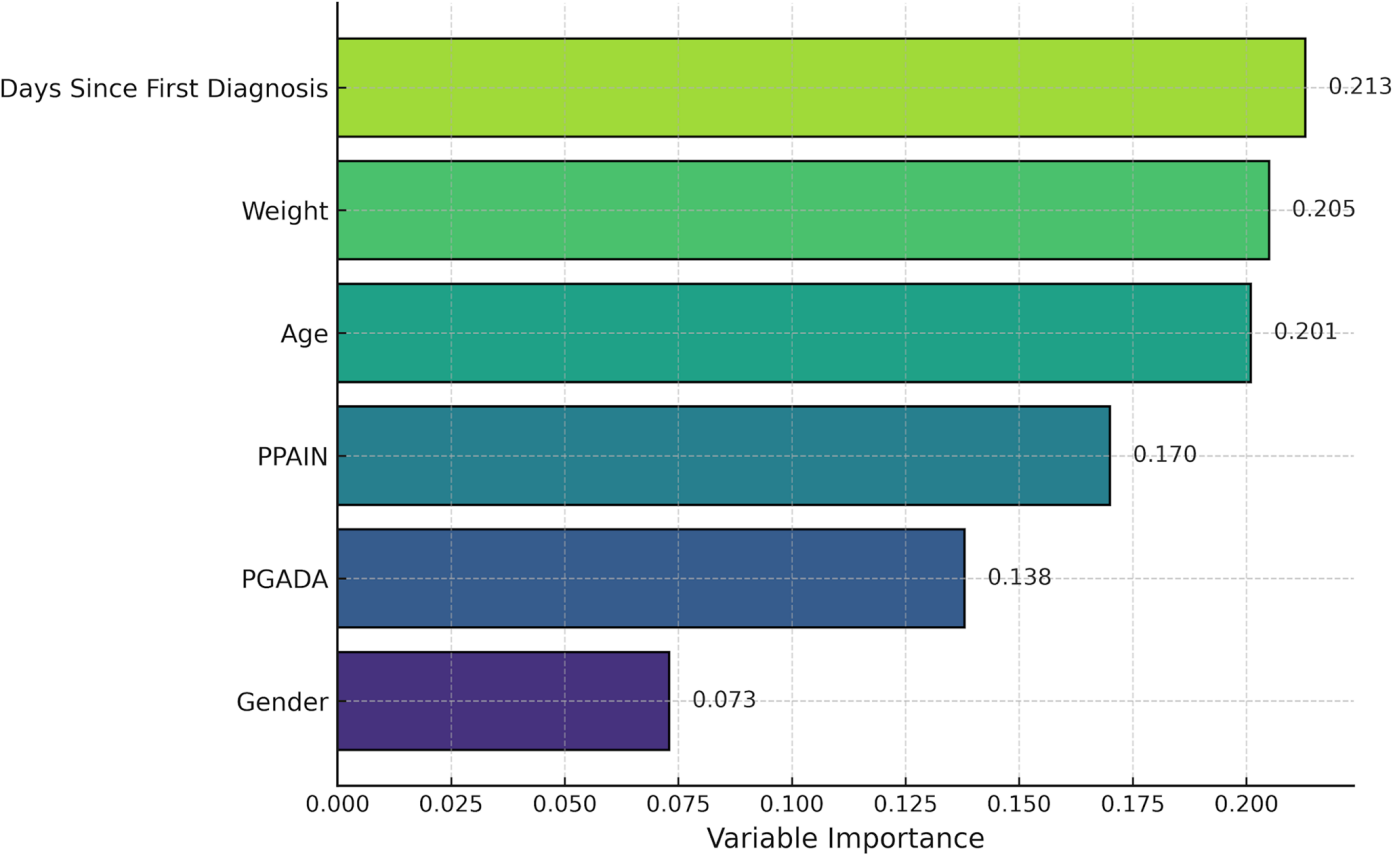
Random Forest feature-importance bar chart. The bar chart displays the relative permutation importance of six predictors used in the final Random Forest classification model (*n* = 2,036). Disease duration contributed the most (21.3%), followed by baseline body weight (20.5%) and age (20.1%). Symptom severity, captured by PPAIN (17.0%) and PGADA (13.8%), also played a meaningful role. Gender showed minimal predictive value (7.3%). PGADA – Patient’s Global Assessment of Disease Activity, PPAIN – Patient’s Global Assessment of Pain Intensity

In the final RF model utilizing six predictors (Fig. 1), disease duration emerged as the most significant determinant of 12-week adherence, accounting for approximately 21.3% of the total variable importance. Patients with a longer time since diagnosis were more likely to remain engaged with the application. Baseline body weight contributed modest predictive information (20.5%; a higher baseline weight was associated with greater adherence) in the multivariable non-linear model, despite no significant mean difference between groups (Table 1). Age also contributed significantly (20.1%), with older individuals more likely to be adherent. Among clinical variables, PPAIN and PGADA showed moderate but comparable importance (17.0% and 13.8%, respectively). Adherence peaked at moderate-to-high PGADA/PPAIN levels and declined at very low or very high levels, consistent with non-linear (inverted-U) patterns in binned analyses and PDPs. Finally, gender contributed minimally (7.3%), with slightly higher adherence observed among female patients; however, the effect was small and likely reflects limited predictive value in clinical decision-making.

**Fig. 2:**
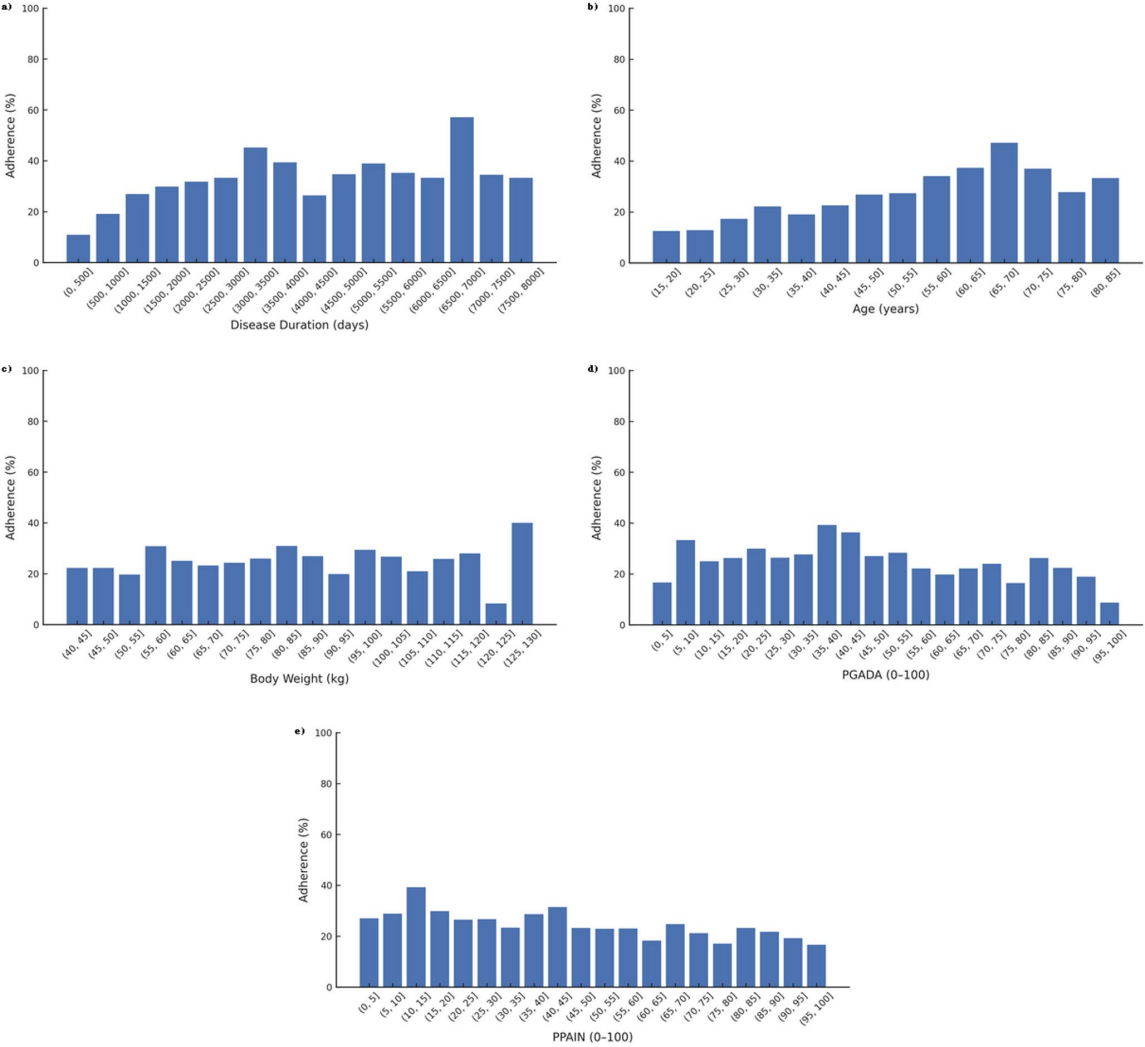
Adherence rates across binned values of clinical and demographic predictors (panels a–e). This figure displays adherence rates across binned values of five key predictors among 2,036 patients. Each subplot represents a distinct variable: (a) Disease duration: adherence increases across early bins and peaks at 3,001–3,500 days (45.2%); (b) Age: Steady rise with a peak in the 62–89 year group (43.4%); (c) Body weight: Peaks at 81–86 kg (31.0%), with moderate variability; (d) PGADA: Inverted-U pattern, peaking at 35–40 (39.3%) and lowest at 95–100 (8.8%); (e) PPAIN: Peak adherence at 10–15 (39.3%), with lowest at 95–100 (16.7%). Per-bin counts and Exact Wilson 95% CIs are reported in Supplementary Tables S4-S8. PGADA – Patient’s Global Assessment of Disease Activity, PPAIN – Patient’s Global Assessment of Pain Intensity

Permutation importance and SHAP indicated the same qualitative ranking of predictors: disease duration was the most influential, followed by baseline weight and age; PtGADA and PPAIN contributed moderately; gender had the smallest impact. SHAP dependence plots showed monotonic, threshold-like increases in predicted adherence with longer disease duration and older age, while PtGADA and PPAIN exhibited non-linear (inverted-U) patterns, consistent with the bin-wise descriptive analyses (Fig. 2, Supplementary Fig. S5–S7). These findings support a combined role of demographic and baseline symptom burden in maintaining adherence over 12 weeks.

Across diagnosis subgroups, the direction of the main associations was consistent; however, subgroup models were underpowered for precise estimates.

Analysis of adherence rates revealed distinct nonlinear patterns across all examined predictors (Fig. 2). For disease duration, adherence increased progressively, reaching its highest value of 45.2% in the 3,001–3,500-day category, whereas the lowest rate of 10.9% was observed among newly diagnosed individuals (0–500 days). Regarding age, adherence rose steadily across deciles, peaking at 43.4% in the 62–89-year group, while the youngest participants (18–28 years) exhibited the lowest adherence at 15.5%. In terms of weight, the highest adherence rate of 31.0% was seen in the 81–86 kg range, compared with the lowest value of 21.7% in the 63–67 kg group. For PGADA scores, adherence was highest (39.3%) in the 35–40 range and lowest (8.8%) in patients with scores between 95 and 100. A similar pattern was observed for PPAIN, with peak adherence of 39.3% in the 10–15 range, declining to 16.7% in those with the most severe pain levels (95–100). Per-bin counts and Wilson 95% CIs are provided in Supplementary Tables S4-S8.

## Discussion

To our knowledge, this is the largest real-world study of digital health application adherence in rheumatology. In a nationwide real-world cohort (*n* = 2,036), older age, longer disease duration, and moderate–high baseline symptom burden (PGADA/PPAIN) were associated with 12-week adherence to the app. These patient characteristics can guide targeting and tailoring of DHIs, while the modest discrimination of our model indicates room for enhancement via richer behavioral data. In line with TRIPOD, we used repeated stratified 5 × 2 CV with out-of-fold CIs, reported full discrimination/calibration and threshold metrics, addressed class imbalance, and added model-agnostic explainability (permutation importance, SHAP), with calibration/PR/DCA shown in the Supplement. Given MAR/MNAR missingness, we applied median imputation. Binning was purely descriptive, as the RF captured nonlinearity without arbitrary cut points.

In line with previous results [10], gender was not a significant predictor in our model. Future implementations of DHA strategies may hence benefit from focusing on patients with longer disease durations and those with stronger baseline engagement. Additional qualitative and prospective studies are warranted to investigate the behavioral and contextual factors that influence adherence.

These findings were consistent with prior literature across RA and other chronic diseases. For example, in studies of type 2 diabetes and hypertension, older age and longer disease duration have also been positively associated with adherence in self-monitoring and digital health adherence. A study by Kebede and Pischke found that older adults with type 2 diabetes were more likely to engage with mobile health applications and showed higher eHealth literacy scores [45]. Similarly, a digital intervention study in patients with cardiovascular disease demonstrated that individuals with more advanced disease and higher symptom burden showed greater sustained adherence over time [46]. Moreover, a systematic review on mHealth adherence in chronic conditions found that older age, self-efficacy, and symptom perception were consistently associated with better adherence to digital interventions [47].

These parallels suggest that standard behavioral mechanisms such as increased motivation for disease control, stable routines, and perceived benefit may underlie digital adherence across different chronic illness populations, not only within rheumatology.

Our findings are consistent with the study by Stradford et al., which reported higher ePRO completion and smartwatch usage among users aged 65 years or older in the ArthritisPower app (adherence rate 87%) compared to younger participants [5]. Similarly, a six-month RA mobile app study found that older users were more adherent [24].

Although the adherence endpoint is operationalized as a single week-12 weight entry, its co-movement with broader adherence signals (higher module/assessment completion among adherent users) supports its construct validity as a practical marker of continued app use. At the same time, we acknowledge that it does not quantify interaction intensity; therefore, we complemented the analysis with model-agnostic performance, calibration, decision-curve, and threshold summaries to provide a fuller picture of predictive utility (Supplementary Figs. S1–S3; Supplementary Table S1).

There are several plausible interpretations of these results:

Older age: This may reflect a more stable life routine, heightened awareness of health needs, and increased intrinsic motivation for self-care. This aligns with prior findings in RA, diabetes, and cardiovascular conditions [20, 23, 31]. Notably, technological literacy appears to be less decisive than mental motivation and personal goals.

Disease duration: Patients with established inflammatory arthritis often undergo more complex clinical trajectories and adapt to long-term treatment. They may perceive greater value in monitoring, recognize actionable feedback from the data, and thus demonstrate higher adherence [6, 25–27].

Body weight: Group means for baseline weight did not differ significantly (Table 1). However, within the multivariable, non-linear RF, the weight contributed a modest predictive signal (permutation importance; Supplement Fig. S4). We therefore avoid implying a strong independent effect of weight and frame it as ancillary to age/disease duration/PROs.

One possible explanation is the built-in digital education component of the Mida Rheuma App, which includes personalized content to support weight management and healthy diet changes. A recent study demonstrated that DHI targeting dietary behavior in patients with rheumatic diseases led to measurable improvements in nutritional quality [48]. Patients with higher BMI may be more motivated to engage with such tools to improve their overall health, particularly when the app directly addresses weight reduction strategies. A recent real-world study found that weight reduction DHAs were among the most effective and also had some of the highest patient adherence rates [9].

Regarding clinical disease severity moderate-high symptom burden is associated with sustained use; extremes (very low or very high) correspond to lower adherence. Adherence with PRO-based applications may offer psychological reassurance and a sense of control to patients who perceive a moderate-high disease burden. Previous studies have shown that symptom severity is positively associated with digital health engagement, particularly in the context of self-monitoring tools for chronic pain and inflammatory conditions [49]. Moreover, structured patient engagement tools have been linked to improved adherence and satisfaction [50]. Similarly, in rheumatology, ePRO implementations have been associated with improved communication and empowerment [51] and with greater acceptance among patients and healthcare providers [52].

Non-significance of gender: Despite some prior reports suggesting that female patients may exhibit better adherence to pharmacological therapies, gender appears less relevant in the context of digital health [11, 35, 43]. However, the possibility of interaction effects or subgroup-specific roles (e.g., age-gender interaction or diagnosis-specific differences) cannot be excluded based on our current analysis. Future modeling efforts using larger samples or stratified approaches should explore whether gender moderates adherence within specific age groups, disease categories, or adherence profiles. This would allow for a more targeted interpretation, rather than generalizing a limited influence across the entire population.

Our refined stratified analysis confirms threshold effects and nonlinear associations across key predictors of digital health adherence. Adherence rates rose markedly beyond 3,000 days since diagnosis and in older age brackets, supporting the notion that long-term disease experience and stable life routines facilitate sustained digital adherence. Conversely, newly diagnosed and younger patients consistently exhibited lower adherence.

In terms of clinical parameters, moderate levels of symptom burden were associated with the highest adherence. Notably, both PGADA and PPAIN displayed inverted U-shaped patterns, with decreased adherence among patients reporting either minimal or very high symptom intensity. These observations imply that a moderate disease burden may represent an optimal window for engaging patients with digital health tools. In contrast, low or overwhelming symptoms may reduce perceived benefit or hinder participation.

Body weight showed a mild nonlinear pattern, with the highest adherence found in the 81–86 kg bin. This may reflect responsiveness to weight-related modules or self-monitoring features embedded within the intervention.

These findings are consistent with the most recent evidence synthesis in rheumatology. A 2025 systematic review of digital applications for self-management in rheumatic and musculoskeletal diseases concluded that DHAs can support activation and symptom monitoring, but sustained adherence hinges on tailored onboarding, feedback loops, and integration with routine care [53]. Our threshold patterns (older age, > 3,000 days since diagnosis, moderate-to-severe symptom burden) provide concrete targeting heuristics that operationalize these recommendations.

Overall, these updated results reinforce the need for stratified digital strategies that address adherence challenges in younger, newly diagnosed, or symptom-extreme populations.

The strengths of our study include the use of a large, real-world dataset comprising over 2,000 patients with inflammatory arthritis. The final predictive model was developed using an RF algorithm, which allowed for the inclusion of multiple clinically relevant variables (age, gender, time since diagnosis, baseline body weight, pain intensity, and patient global disease activity) despite data heterogeneity and missing values. This approach provided interpretable variable importance scores while ensuring model robustness in the presence of incomplete assessments. The study also demonstrated that routinely collected digital patient-reported outcomes (e.g., pain and global disease activity) can meaningfully contribute to adherence prediction, which highlights the value of patient-generated health data for implementation science in rheumatology.

### Limitations

This retrospective observational design limits causal inference and precludes complete control of confounding. Key behavioral/psychosocial determinants of digital health adherence (education, socioeconomic status, digital literacy, intrinsic motivation, comorbidities) were unavailable, reducing explanatory depth and external validity. Our adherence proxy, presence of a week-12 weight entry, is unidimensional; nonetheless, convergent app-engagement signals (Supplementary Table S1) support its face validity. Clinical disease activity biomarkers (e.g., DAS28, C-reactive protein, corticosteroid use) were not captured, constraining clinical interpretation. Selection bias is possible: users of a certified rheumatology app may be more motivated or health-literate than the broader population, and generalizability is further limited by the Germany/Austria/Switzerland regulatory context. Missingness likely mixed MAR and MNAR mechanisms (e.g., PRO gaps related to observed covariates versus excluded users without baseline weight), so effect sizes should be interpreted cautiously despite transparent flow/missingness reporting. Finally, the model’s discriminative performance was modest (ROC AUC = 0.627), reflecting the complexity of real-world adherence. Future prospective work should incorporate richer behavioral signals (onboarding completion, notifications, session counts), standardized psychosocial measures, and multimodal data (usage telemetry, sensors, ecological momentary assessment) to improve calibration, transportability, and clinical utility.

## Conclusion

This real-world study of over two thousand patients with inflammatory arthritis shows that sustained adherence with a rheumatology digital health app is most likely among older patients, those with longer disease duration, moderate–to–high symptoms, and higher body weight. These insights highlight the need for stratified onboarding and targeted support for patients at risk of disengagement. Future research should integrate behavioral and psychosocial factors to improve prediction and personalization, enabling digital health tools to deliver lasting, patient-centered care in rheumatology.

## Supplementary Information

Below is the link to the electronic supplementary material.

Supplementary Material 1

## Data Availability

Analysis was conducted on anonymized data, under a data-processing agreement, which allowed the authors to maintain independent analytic control. Data Sharing: Midaia GmbH data is not approved for sharing.
