# Supplementary material for: Predictors of adherence to a digital health application in patients with inflammatory arthritis: retrospective analysis
Source: Rheumatol Int. 2025 Dec 15;46(1):16. doi: 10.1007/s00296-025-06017-9 (PMC12705853; doi:10.1007/s00296-025-06017-9)

Table S1. Engagement indicators by adherence group (Baseline cohort)

Engagement indicator	Adherent (n=526)	Non-adherent (n=1510)	p-value
Number of completed therapeutic modules/assessments (As): mean ± SD	7.23 ± 6.98	1.90 ± 1.42	<0.001
Number of completed APs: median [IQR]	5.00 [2.00; 10.00]	1.00 [1.00; 2.00]	
Users with ≥1 completed AP, %	100.0%	100.0%	
Users with ≥4 completed APs, %	60.7%	12.5%	
Users with ≥8 completed APs, %	32.4%	0.0%	

AP - action plan, SD – standard deviation, IQR - interquartile range.

Table S2. Discrimination and calibration (repeated stratified 5×2 CV)

Metric	Estimate	95% CI (if applicable)	Notes
ROC AUC	0.627	0.600–0.654	Out-of-fold predictions (5×2 CV)
PR-AUC	0.359	—	Precision–Recall area under curve
Brier score	0.189	—	Probability calibration (lower is better)

ROC AUC - Receiver Operating Characteristic Area Under Curve, PR AUC - Precision-Recall Area Under Curve, CI - confidence interval

Table S3. Threshold-specific performance (out-of-fold, repeated stratified 5×2 CV)

Threshold	Sensitivity	Specificity	PPV	NPV	F1	Balanced Accuracy
p = 0.50	0.131	0.938	0.424	0.756	0.200	0.534
Youden (p ≈ 0.26)	0.627	0.580	0.342	0.817	0.443	0.604

PPV - positive predictive value, NPV - negative predictive value.

Note. The “F1-optimal” operating point requires the full score distribution across folds; these summary aggregates do not allow exact recovery of that threshold and are thus omitted here.

Table S4. Disease duration bins, adherence and Wilson 95% CIs

Bin	n_adherent	n_total	Adherence %	95% CI (low %)	95% CI (high %)
0–500	26	239	10.9	7.5	15.5
500–1000	57	299	19.1	15.0	23.9
1000–1500	70	260	26.9	21.9	32.6
1500–2000	43	144	29.9	23.0	37.8
2000–2500	35	110	31.8	23.9	41.0
2500–3000	24	72	33.3	23.5	44.8
3000–3500	42	93	45.2	35.4	55.3
3500–4000	24	61	39.3	28.1	51.9
4000–4500	10	38	26.3	15.0	42.0
4500–5000	17	49	34.7	22.9	48.7
≥5001	114	335	34.0	29.2	39.3

CI - confidence interval

Table S5. Age bins, adherence and Wilson 95% CIs

Bin	n_adherent	n_total	Adherence %	95% CI (low %)	95% CI (high %)
(13, 20]	2	16	12.5	3.5	36.0
(20, 27]	27	165	16.4	11.5	22.8
(27, 34]	55	279	19.7	15.5	24.8
(34, 41]	74	352	21.0	17.1	25.6
(41, 48]	78	344	22.7	18.6	27.4
(48, 55]	102	372	27.4	23.1	32.2
(55, 62]	102	311	32.8	27.8	38.2
(62, 69]	64	136	47.1	38.9	55.4
(69, 76]	14	43	32.6	20.5	47.5
(76, 83]	6	16	37.5	18.5	61.4
(83, 90]	1	1	100.0	20.7	100.0

CI - confidence interval

Table S6. Weight bins, adherence and Wilson 95% CIs

Bin	n_adherent	n_total	Adherence %	95% CI (low %)	95% CI (high %)
(40, 45]	2	9	22.2	6.3	54.7
(45, 50]	10	45	22.2	12.5	36.3
(50, 55]	21	107	19.6	13.2	28.1
(55, 60]	53	172	30.8	24.4	38.1
(60, 65]	54	215	25.1	19.8	31.3
(65, 70]	53	228	23.2	18.2	29.1
(70, 75]	55	226	24.3	19.2	30.3
(75, 80]	54	208	26.0	20.5	32.3
(80, 85]	60	194	30.9	24.8	37.7
(85, 90]	46	171	26.9	20.8	34.0
(90, 95]	24	121	19.8	13.7	27.8
(95, 100]	30	102	29.4	21.4	38.9

(100, 105]	20	75	26.7	18.0	37.6
(105, 110]	9	43	20.9	11.4	35.2
(110, 115]	8	31	25.8	13.7	43.2
(115, 120]	7	25	28.0	14.3	47.6
(120, 125]	1	12	8.3	1.5	35.4
(125, 130]	6	15	40.0	19.8	64.3

CI - confidence interval

Table S7. PtGADA bins, adherence and Wilson 95% CIs

Bin	n_adherent	n_total	Adherence %	95% CI (low %)	95% CI (high %)
0–5	11	49	22.4	13.0	35.9
6–10	21	63	33.3	22.9	45.6
11–15	12	48	25.0	14.9	38.8
16–20	21	80	26.2	17.9	36.8
21–25	18	60	30.0	19.9	42.5
26–30	24	91	26.4	18.4	36.3
31–35	13	47	27.7	16.9	41.8
36–40	42	107	39.3	30.5	48.7
41–45	12	33	36.4	22.2	53.4
46–50	26	96	27.1	19.2	36.7
51–55	23	81	28.4	19.7	39.0
56–60	33	149	22.1	16.2	29.5
61–65	21	106	19.8	13.3	28.4
66–70	52	235	22.1	17.3	27.9
71–75	43	179	24.0	18.4	30.8
76–80	34	207	16.4	12.0	22.1
81–85	31	118	26.3	19.2	34.9
86–90	26	116	22.4	15.8	30.8
91–95	7	37	18.9	9.5	34.2
96–100	5	57	8.8	3.8	18.9

CI - confidence interval

Table S8. PPAIN bins, adherence and Wilson 95% CIs

Bin	n_adherent	n_total	Adherence %	95% CI (low %)	95% CI (high %)
0–5	18	61	29.5	19.6	41.9
6–10	24	83	28.9	20.3	39.4
11–15	22	56	39.3	27.6	52.4
16–20	31	104	29.8	21.9	39.2
21–25	18	68	26.5	17.4	38.0
26–30	27	101	26.7	19.1	36.1
31–35	14	60	23.3	14.4	35.4
36–40	35	122	28.7	21.4	37.3
41–45	17	54	31.5	20.7	44.7
46–50	23	99	23.2	16.0	32.5

51–55	22	96	22.9	15.6	32.3
56–60	35	152	23.0	17.0	30.3
61–65	25	137	18.2	12.7	25.6
66–70	54	218	24.8	19.5	30.9
71–75	33	156	21.2	15.5	28.2
76–80	26	152	17.1	11.9	23.9
81–85	20	86	23.3	15.6	33.2
86–90	20	92	21.7	14.5	31.2
91–95	5	26	19.2	8.5	37.9
96–100	6	36	16.7	7.9	31.9

CI - confidence interval

Supplement Fig. S1 Calibration curve. Calibration plot from out-of-fold predictions of the RF model (repeated stratified 5-fold CV, 2 repeats); dashed line denotes perfect calibration.

Supplement Fig. S2 Precision–Recall curve. PR curve from OOF predictions; dashed horizontal line marks outcome prevalence (adherent rate).

Supplement Fig. S3 Decision Curve Analysis. Net benefit across threshold probabilities (0.05–0.90) for the RF model using OOF predictions.

Supplement Fig. S4 Permutation importance. Hold-out–based permutation importance (mean AUC drop over 20 permutations per feature; 80/20 stratified split) for six predictors (Days Since First Diagnosis, Age, PGADA, PPAIN, Weight, Gender).

Supplement Fig. S5 SHAP summary (beeswarm) for the six-predictor Random Forest model. Each point represents one patient; the horizontal position indicates the SHAP value (impact on the predicted probability of 12-week adherence), and the color encodes the underlying feature value (from low to high). Positive SHAP values indicate a higher predicted likelihood of adherence.

Supplement Fig. S6 SHAP dependence plot for Days Since First Diagnosis Date. The curve illustrates the non-linear relationship between disease duration and the predicted probability of 12-week adherence, with point color indicating the corresponding feature value. Higher duration is associated with a larger positive impact, consistent with the threshold effects observed in the binned analysis.

Supplement Fig. S7 SHAP dependence plot for Age. The plot illustrates a monotonic, non-linear relationship where older age contributes positively to predicted adherence, with diminishing returns at the upper tail.

Supplement Fig. S1 Calibration curve.

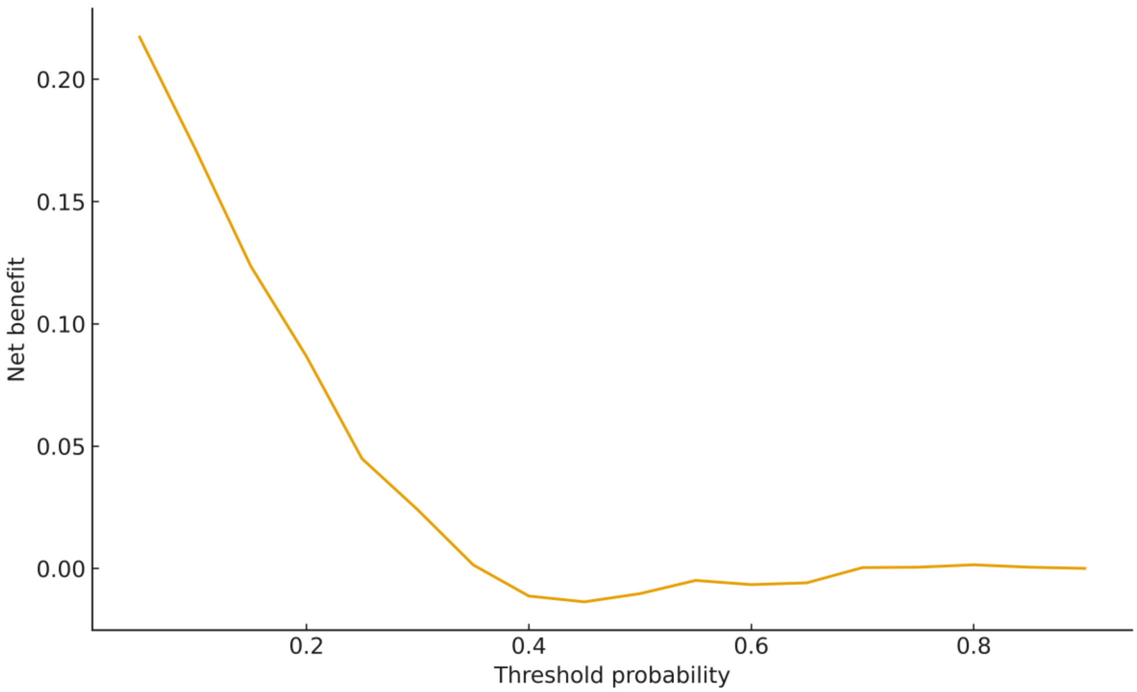

Supplement Fig. S2 Precision–Recall curve.

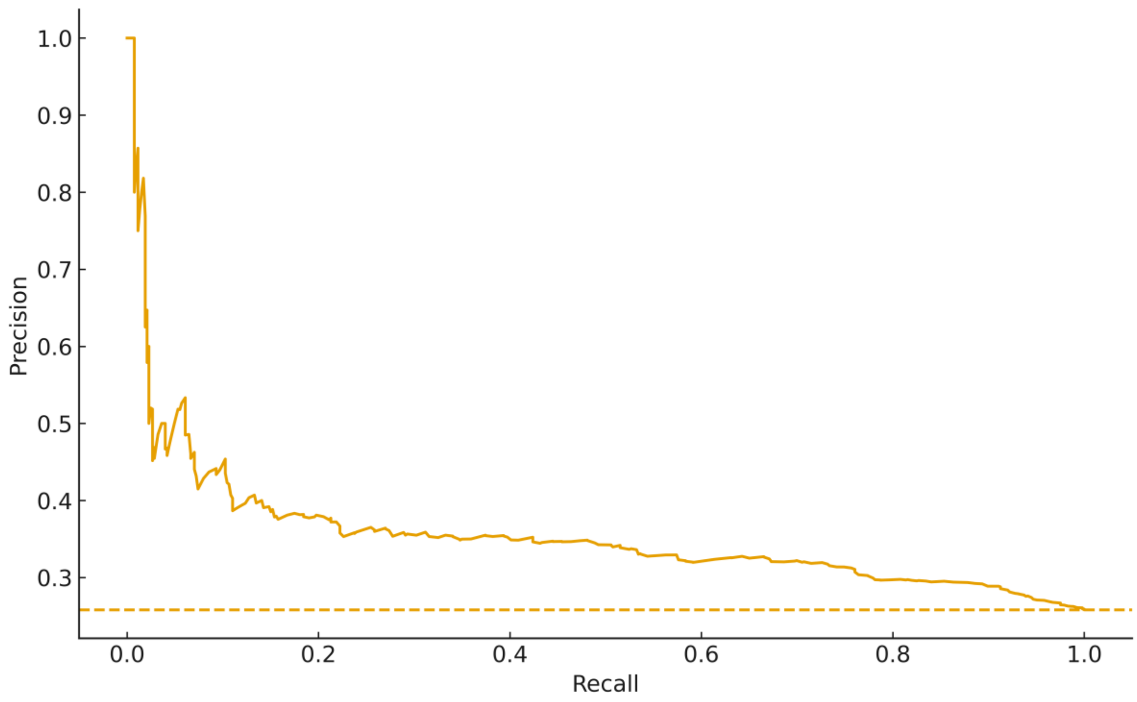

Supplement Fig. S3 Decision Curve Analysis.

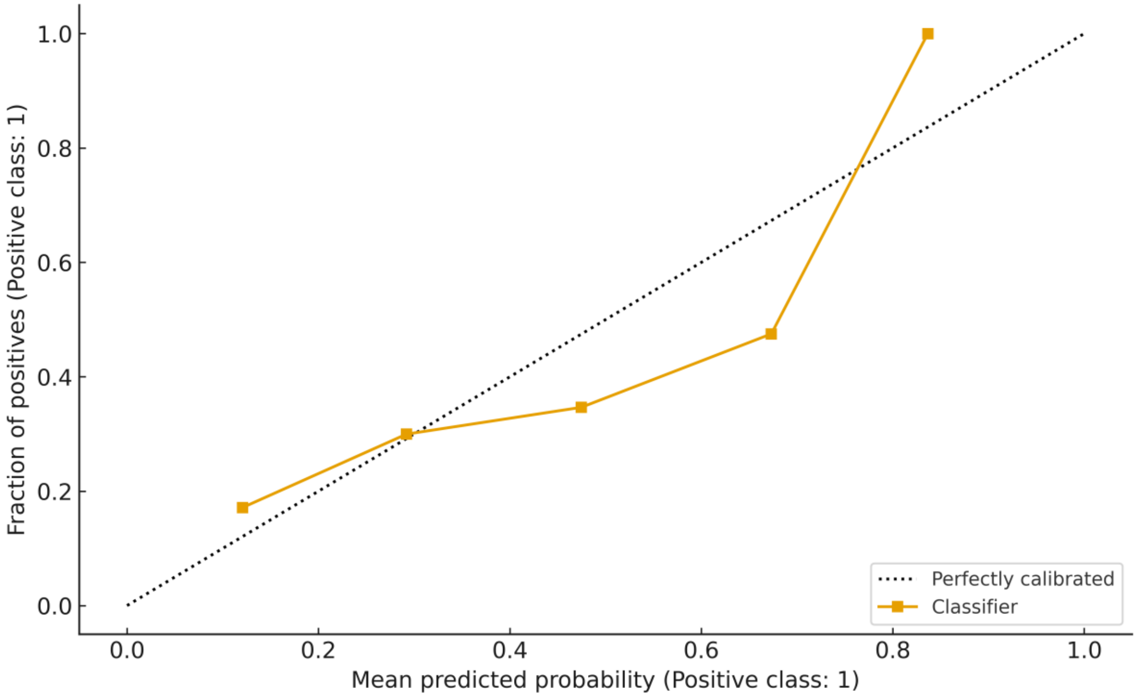

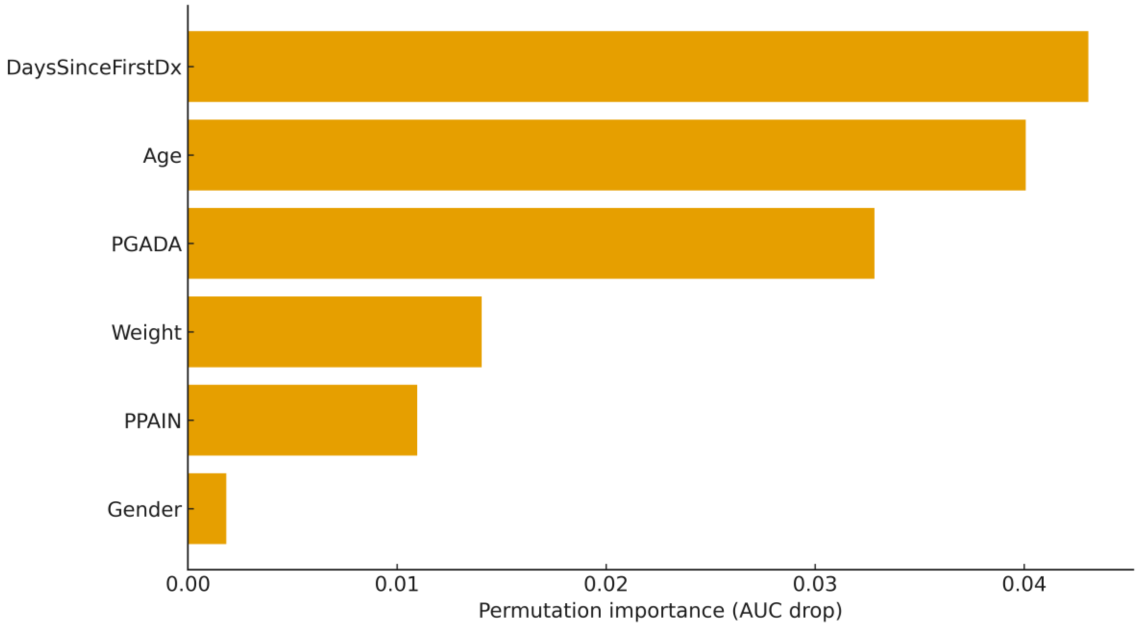

Supplement Fig. S5 SHAP summary (beeswarm) for the six-predictor Random Forest model.

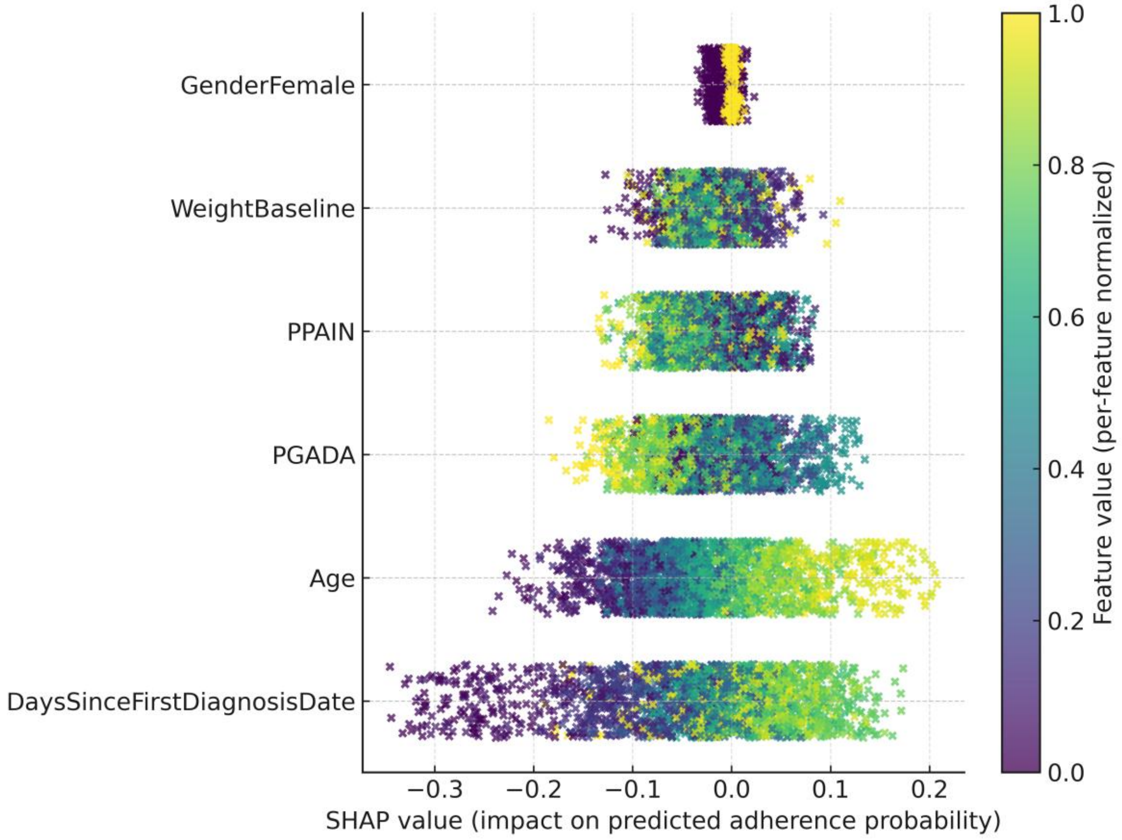

Supplement Fig. S6 SHAP dependence plot for Days Since First Diagnosis Date.

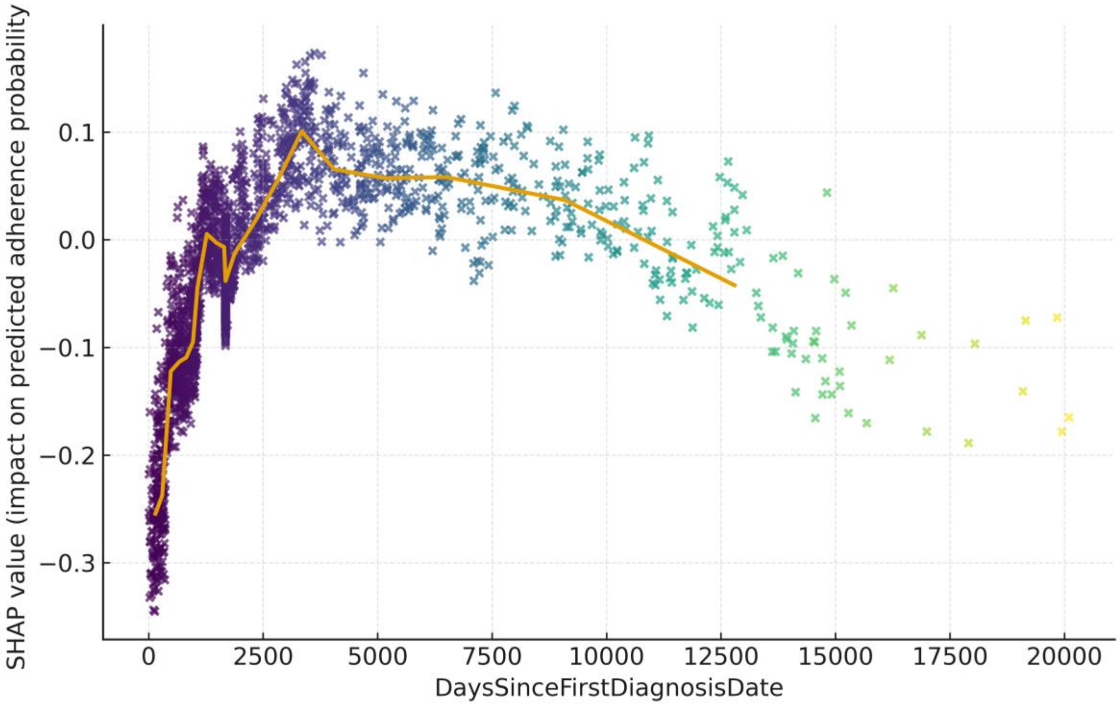

Supplement Fig. S7 SHAP dependence plot for Age.

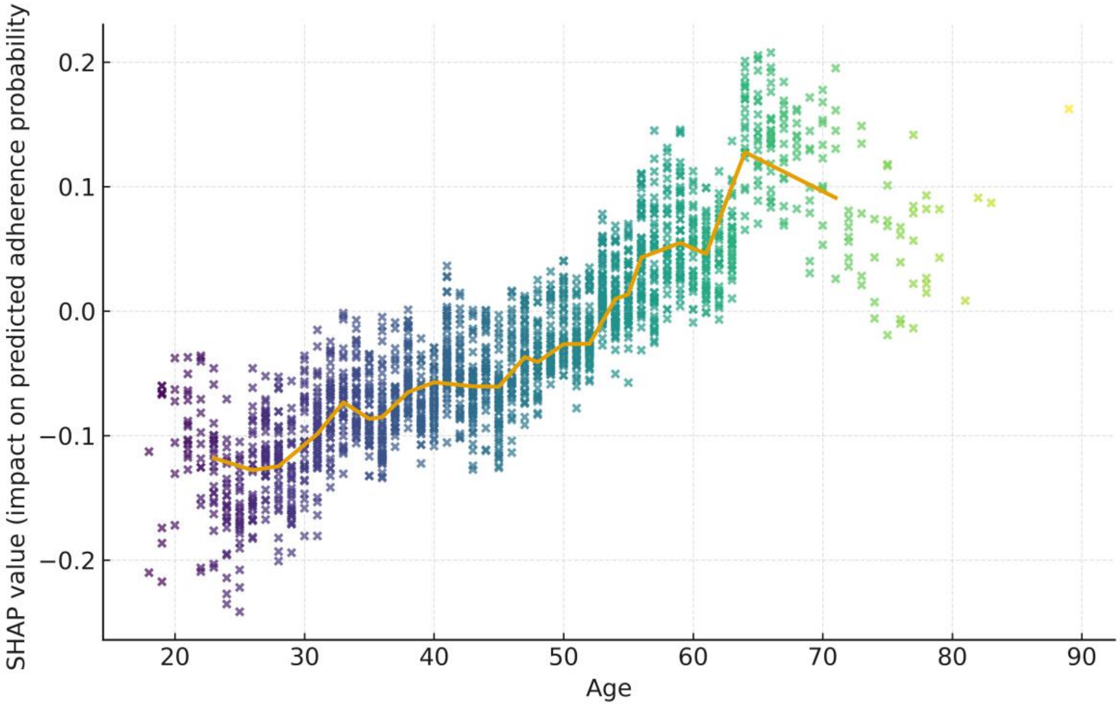

Supplement: Supplementary file 1 — Supplementary Material 1 [file 296_2025_6017_MOESM1_ESM.pdf]
